# Tetraploid embryonic stem cells can contribute to the development of chimeric fetuses and chimeric extraembryonic tissues

**DOI:** 10.1038/s41598-017-02783-0

**Published:** 2017-06-08

**Authors:** Bingqiang Wen, Ruiqi Li, Keren Cheng, Enhong Li, Shaopeng Zhang, Jinzhu Xiang, Yanliang Wang, Jianyong Han

**Affiliations:** 1 State Key Laboratory for Agro biotechnology, College of Biological Sciences, China Agricultural University, Beijing, 100193 People’s Republic of China; 2 Reproductive Medicine Centre, Department of Obstetrics and Gynecology, Sun Yat-Sen Memorial Hospital, Sun Yat-Sen University, Guangzhou, 510120 People’s Republic of China; 3 Department of Biology, The University of Texas at San Antonio, UTSA one Circle, San Antonio, TX 78249 United States

## Abstract

Our study examined the *in vivo* chimeric and survival capacities of chimeras created by injecting tetraploid embryonic stem cells (ESCs) expressing green fluorescent protein (GFP) into diploid embryos. At 3.5 days post-coitum (dpc) and 4.5 dpc, the tetraploid ESCs were able to contribute to the inner cell mass (ICM) just as diploid ESCs tagged with GFP. At 6.5 dpc, 8.0 dpc and 10.5 dpc, the tetraploid ESCs manifested in the same location as the diploid ESCs. The GFP cells in the extraembryonic tissues and fetuses of tetraploid ESC chimeras were tetraploid as determined by fluorescence activated cell sorting (FACS). Furthermore, tetraploid ESCs contributed to the development of the placenta, embryolemma and umbilical cord at 13.5 dpc and 16.5 dpc; however, very less GFP cells were found in the fetuses of tetraploid ESC chimeras. We further found that the proliferation of tetraploid ESCs was slower than that of diploid ESCs. In addition, the relative mRNA expression in the three germ layers and the trophoblast was abnormal in the EBs of tetraploid ESCs compared with diploid ESCs. In short, slower proliferation and abnormal differentiation potential of tetraploid ESCs might be two of the reasons for their poor survival and chimeric capacities.

## Introduction

Tetraploid cells can be made by fusing two diploid cells regardless of the cell cycle stage. Previous studies used polyploid mammalian cells to investigate cell growth and cytogenetic changes^1^. Somatic cell fusion with ESCs to form a hybrid cells has provided an approach to study the mechanism of how the cell reprogramming occurs. Tetraploid hybrid cells exhibited a similar cell cycle as ESCs and shared immortal growth characteristics and cell markers as ESCs^2^. There are various ways to produce tetraploid cells *in vitro*—pluripotent tetraploid hybrid cells can be generated from the fusion of somatic cells with ESCs^2, 3^, embryonic germ cells^4^ or embryonic carcinoma cells^5^. Homozygous tetraploid ESCs derived from parthenogenetic tetraploid blastocysts exhibited high pluripotent capacity and were able to form both chimeras during the blastocyst stage and teratomas containing derivatives of the three germ layer cells^6^, suggesting that polyploidization of tetraploid ESCs is normal during the early stages of development. However, the postimplantation development of homozygous tetraploid ESCs chimeric embryos is still unknown.

Mouse tetraploid embryos were successfully generated by fusing two blastomeres within the zona pellucida and can also be produced by injecting somatic cells into intact MII oocytes^7^. A previous study of tetraploid mouse embryo development showed that tetraploid embryos can undergo compaction and form cavities similar to diploid embryos^8^; furthermore, tetraploid mouse embryos could be recovered on the 15th day of gestation, but their development was retarded^9^. Tetraploid mouse embryos died at various developmental stages. The combination of tetraploid and diploid embryonic cells is known to lead to the development of the entire fetus from either diploid cells of the inner cell mass or diploid culture-derived ESCs^8, 10, 11^. After injection into tetraploid blastocysts, the diploid ESCs give rise to the epiblast, whereas the tetraploid host cells only develop into extraembryonic tissues^10, 12^. This technology, which is designated as “ES tetraploid complementation”^10, 12, 13^, has been successfully applied to the assessment of developmental potential in ESCs derived from nuclear transfer blastocysts^14^ and in induced pluripotent stem (iPS) cells^15^. Unsurprisingly, tetraploid hybrid cells marked by the *lacZ* gene reporter and injected into diploid blastocysts were revealed as providing a single cell contribution in eight out of twenty embryos at 7.5 dpc^3^; this poor contribution of the hybrid cells has been explained as “a severe loss tetraploid cells in the chimeras of diploid and tetraploid embryos”^3^. In the light of these data, reports of the birth of chimeras generated by the injection of hybrid cells with a tetraploid karyotype into diploid blastocysts appear remarkable^16–18^.

However, previous studies were mainly focused on the development of tetraploid embryos and hybrid tetraploid ESCs in chimeric embryos, and little is known about the postimplantation development of homozygous tetraploid ESC-based chimeric embryos.

In humans, single blastomere biopsies during human-assisted reproduction techniques (ART) have frequently revealed polyploidy (triploidy, tetraploidy, and higher order ploidies) or mosaically polyploid preimplantation embryos^19, 20^. The embryos (whether mosaic or total polyploidy) are usually discarded or preferentially not used and are a significant cause for embryonic wastage during early human postimplantation development^21, 22^. However, there are significant questions of whether these embryos with abnormal chromosomal blastomeres could participate in the formation of the fetus or extraembryonic tissues, and their chimeric and survival capacity is unclear. In the current study, tetraploid ESCs were injected into the diploid embryos to simulate human mosaic embryos that have tetraploid epiblast cells. The mouse epiblast has been unequivocally identified as a source of ESCs by means of microsurgical separation from trophoblasts and hypoblasts prior to culture, and these ESCs can produce a chimeric mouse^23^. The epiblast generated an entire fetus and individual mouse epiblast cells, which were isolated at this stage and microinjected into another blastocyst, and can contribute to all lineages of the fetus^24^. Thus, the consequence of total and mosaic embryonic polyploidy are of significant clinical and biological interest.

To study the chimeric and survival capacity of tetraploid ESC chimeras *in vivo*, the tetraploid ESC chimeras were used as a model to infer the activity of human mosaically polyploidy preimplantation embryos. Here, we established GFP-tagged tetraploid ESCs lines *in vitro* and injected these tetraploid ESCs into diploid mouse embryos at the 4–8 cell stage to form chimeric embryos, which were then transferred to pseudocyesis mice. Chimeras were harvested and examined at different developmental stages.

## Results

### Comparison of the blastocyst proportion, outgrowth formation and ESC line establishment between diploid and tetraploid embryos

**Figure 1:**
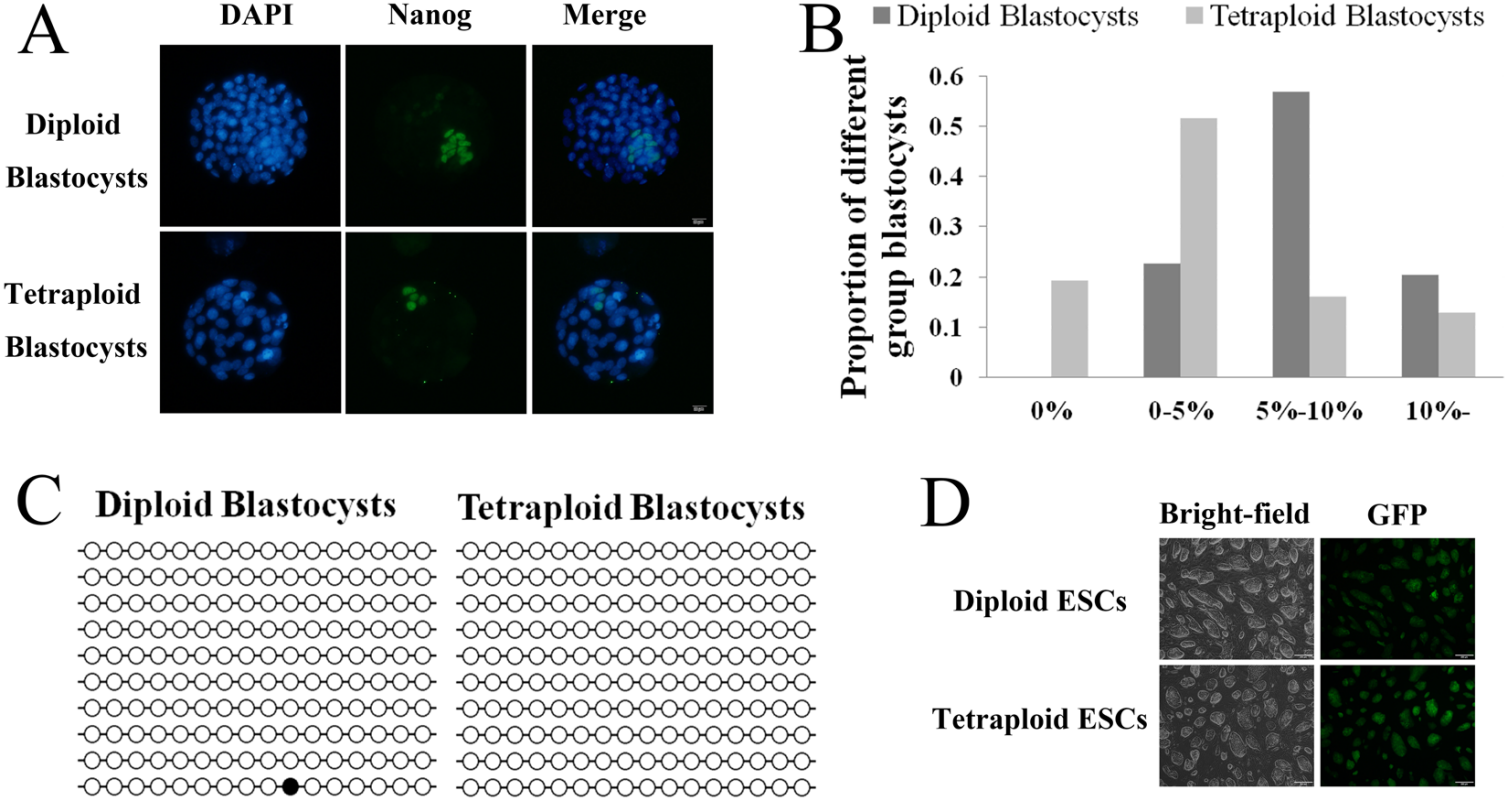
Characterization of 4.5 dpc tetraploid blastocysts. (**A**) Differential expression of NANOG in diploid and tetraploid embryos at the 4.5 dpc blastocyst stage. Bar scale = 20 μm. (**B**) Percentages of different groups of blastocysts. Diploid blastocysts and tetraploid blastocysts were differentially stained with EPI, and total cells were individually classified into four groups according to the number of EPI-positive cells (0%, 0–5%, 5–10% and 10%). 0% represented no epiblast cells in the blastocyst, 0–5% represented the percentage of epiblast cells in the blastocyst greater than 0% and less than or equal to 5%. 5–10% represented the percentage of epiblast cells in the blastocyst greater than 5% and less than or equal to 10%. 10%- represented the percentage of epiblast cells in the blastocyst greater than 10%. (**C**) The methylation status of the *Oct4* promoter showed differentially methylated regions in diploid and tetraploid blastocysts. (**D**) The colonies of diploid and tetraploid ESCs were round and three-dimensional. Bar scale = 200 μm.

Diploid and tetraploid embryos could develop into blastocysts (Table 1); however, the total number of cells stained by DAPI and epiblast cells identified by NANOG in the tetraploid blastocysts was significantly less than those of diploid blastocysts (Table 2 and Fig. 1A). In addition, the percentage of epiblast cells among the total cell population of most tetraploid blastocysts was 0–5%, and the percentage of epiblast cells among the total cell population of most diploid blastocysts was 5–10% (Fig. 1B). The efficiency of the tetraploid blastocyst outgrowth formation was lower than that of diploid blastocysts (46.43% vs 85.71%) (Table 1), but the efficiency of ESC line establishment was similar between diploid and tetraploid outgrowth (100% vs 92.31%) (Table 1). The *Oct4* promoter region methylation levels of the 4.5 dpc diploid and tetraploid blastocysts were both low (Fig. 1C). In addition, the two types of ESCs morphology were similar (Fig. 1D).

**Table 1:** The percentage of developing blastocysts, outgrowth formation and ESC line establishment of diploid and tetraploid embryos.

Type of embryo	No. of embryos	No. of blastocysts	No. with outgrowth (%)	No. of ESC lines (%)
Diploid embryos	28	28 (100%)^a^	24 (85.71%)^a^	24 (100%)^a^
Tetraploid embryos	28	28 (100%)^a^	13 (46.43%)^b^	12 (92.31%)^a^

*Values with different letters within the same column are significantly different (*P* < 0.05).

**Table 2:** The number of total cells and epiblast cells in 4.5 dpc diploid and tetraploid blastocysts.

Type of embryo	No. of blastocysts	No. of total cells	No. of EPI cells
Diploid embryos	43	93.73 ± 14.77^a^	7.2 ± 2.73^a^
Tetraploid embryos	33	47.65 ± 8.13^b^	2.23 ± 2.03^b^

^*^Values with different letters within the same column are significantly different (*P* < 0.05).

### The tetraploid ESCs karyotype and pluripotency analysis

**Figure 2:**
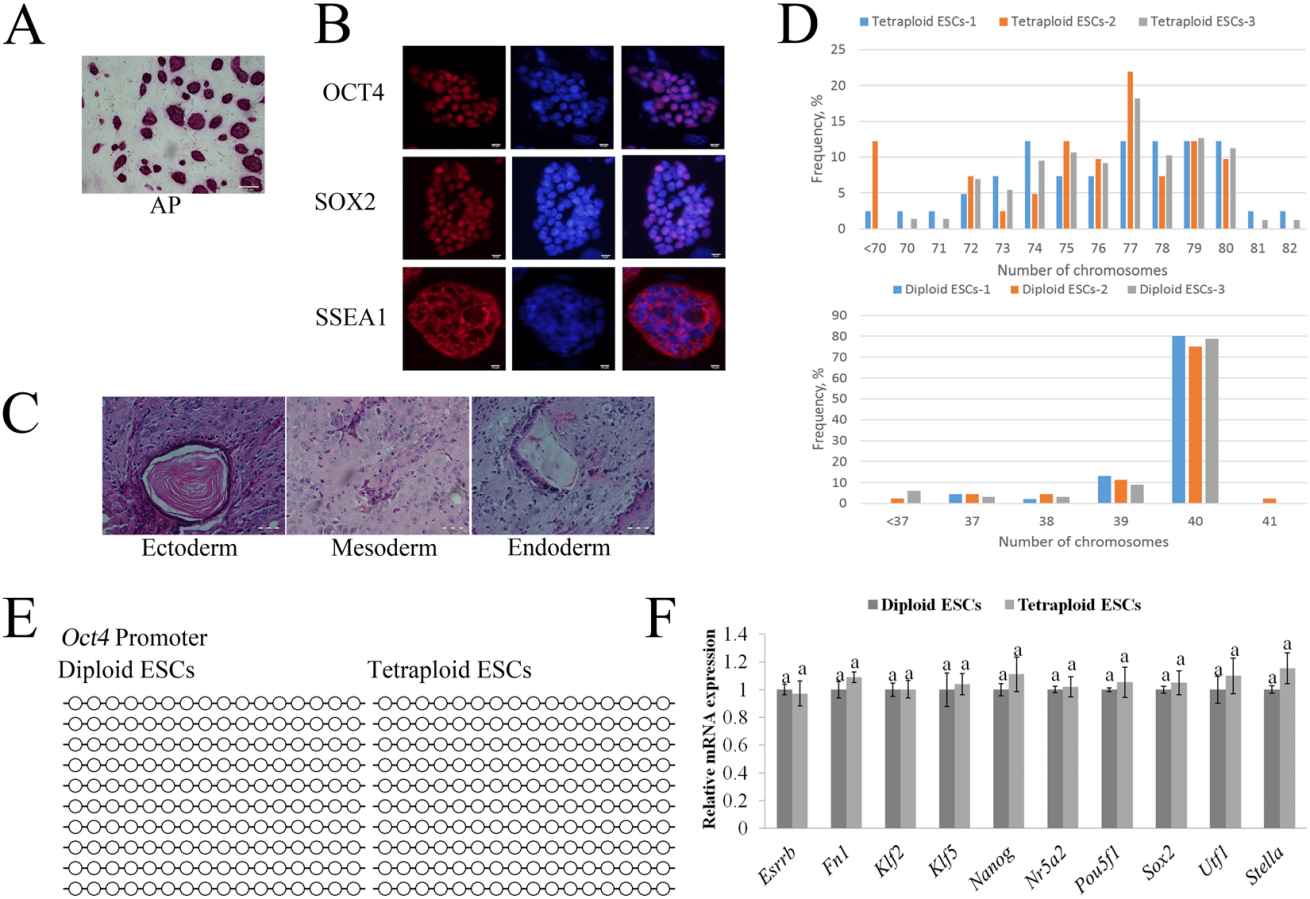
Characterization of tetraploid ESCs. (**A**) Tetraploid ESCs were positive for AP staining. Bar scale = 200 μm. (**B**) Immunocytochemical staining of OCT4, SOX2 and SSEA1 in tetraploid ESCs. Bar scale = 10 μm. (**C**) Hematoxylin and eosin staining of teratoma sections of tetraploid ESCs. Left: blood vessel of endothelium (ectoderm); middle: muscle (mesoderm); right: gut-like epithelium (endoderm). Scale bars = 50 μm. (**D**) Karyotype analysis of diploid and tetraploid ESCs. (**E**) Methylation status of the *Oct4* promoter showed methylated regions in diploid and tetraploid ESCs. (**F**) Relative mRNA expression levels of pluripotent genes in diploid and tetraploid ESCs. Different superscripts represent statistically significant differences between groups (*P* < *0*.*05*).

Tetraploid ESCs stained AP-positive (Fig. 2A), and the tetraploid ESCs expressed diploid ESC pluripotent markers, including the transcription factors OCT4, SOX2 and SSEA1 (Fig. 2B). Tetraploid ESCs developed into teratomas in immunodeficient mice. Hematoxylin and eosin staining suggested that these teratomas contained three germ layer cells (Fig. 2C). However, the chromosome number of the tetraploid ESCs was disordered and only about 15% were normal (Fig. 2D). In addition, the *Oct4* (*pou5f1*) promoter region methylation levels of diploid and tetraploid ESCs were at low levels (Fig. 2E). In addition, there were no significant differences in the relative expression of pluripotent genes (e.g., *Esrrb*, *Fn1*, *Klf2*, *Klf5*, *Nanog*, *Nr5a2*, *Pou5f1*, *Sox2*, *Utf1* and *Stella*) between the diploid and tetraploid ESCs as determined by quantitative real-time PCR (Fig. 2F).

### Analysis of the developmental capacity of chimeras formed by the injection of the two types of ESCs into diploid embryos

**Figure 3:**
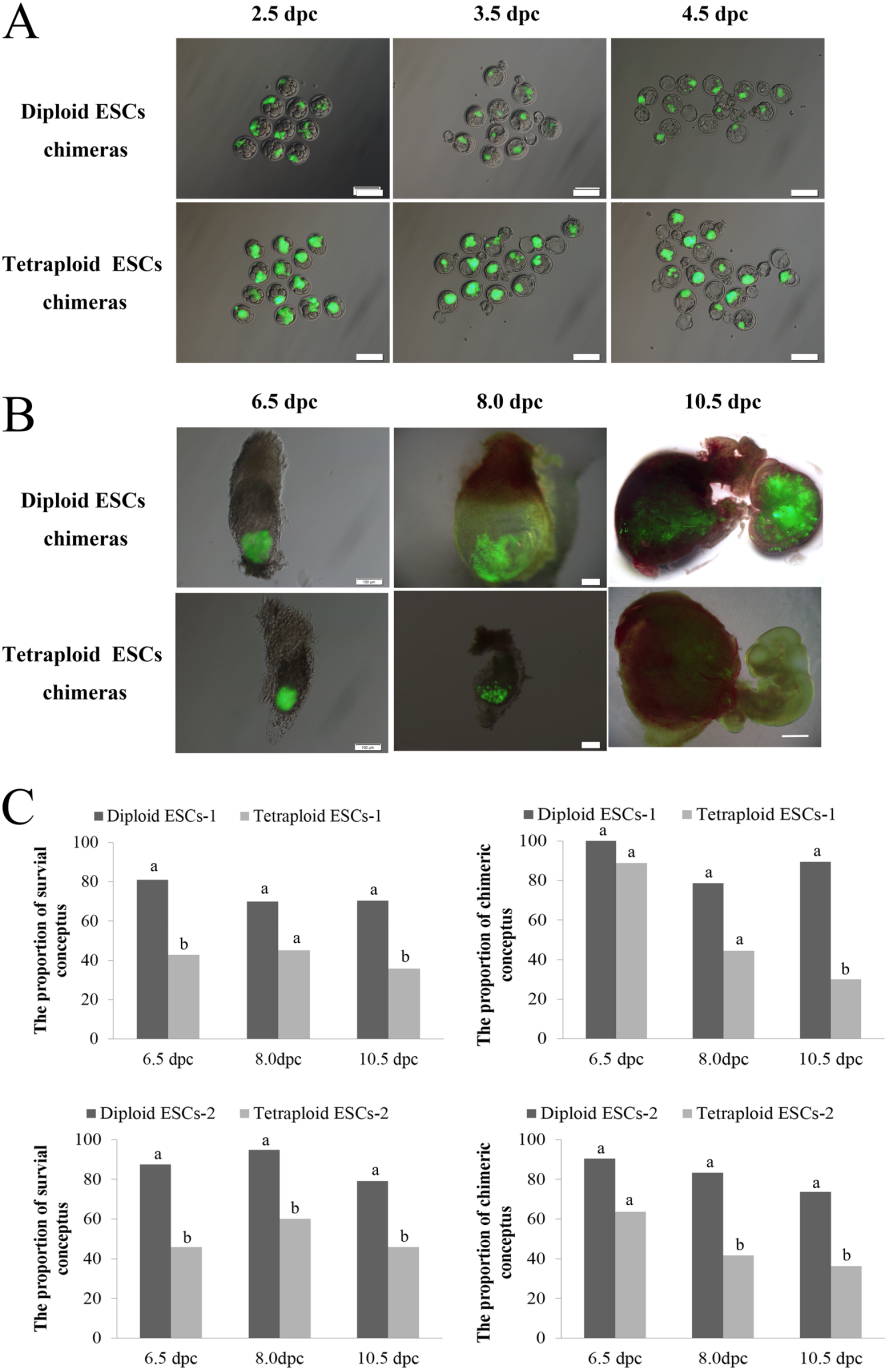
The chimeric capacity of diploid and tetraploid ESCs. (**A**) Diploid ESC and tetraploid ESC chimeric embryos at 2.5 dpc, 3.5 dpc and 4.5 dpc. Bar scale = 100 μm. (**B**) Diploid ESC and tetraploid ESC chimeras at 6.5 dpc (Bar scale = 100 μm), 8.0 dpc (Bar scale = 200 μm) and 10.5 dpc (Bar scale = 2 mm). (**C**) The percentage of survival conceptus and chimeric conceptus at 6.5 dpc, 8.0 dpc and 10.5 dpc. Different superscripts represent statistically significant differences between groups (*P* < .05).

Both diploid and tetraploid ESCs were chimeric in the ICM at 3.5 dpc and 4.5 dpc blastocysts when 15 cells from either diploid or tetraploid ESCs were injected into 4–8 cell embryos (Fig. 3A and Supplementary Table S1–1, S1–2). The tetraploid ESC chimeras grew slower. At 6.5 dpc, the tetraploid ESC chimeras were abnormal, and the chimeric capacity and chimeras’ survival capacity were lower than the chimeras formed by injection of diploid ESCs into diploid embryos (Fig. 3B,C, Supplementary Fig. S3A and Supplementary Table S2–1, S2–2). At 8.0 dpc, the chimeric capacity and chimeras’ survival capacity were lower than the diploid ESC chimeras, just as 6.5 dpc (Fig. 3B,C, Supplementary Fig. S3A and Supplementary Table S3–1, S3–2). At 10.5 dpc, tetraploid cells derived from tetraploid ESCs were chimeric in the fetus and the extraembryonic tissues (i.e., placenta, embryolemma and umbilical cord), just as the diploid chimera (Fig. 3B and Supplementary Fig. S3A). However, compared with the diploid ESC chimeras, the survival chimeric capacities of tetraploid ESC chimeras were significantly poorer than those of diploid ESC chimeras (Fig. 3C and Supplementary Table S4–1, S4–2). In general, the chimeric capacity of tetraploid ESCs declined, and the survival capability was poor (Fig. 3C). At 10.5 dpc, the GFP-labeled cells in the fetus and extraembryonic tissues of tetraploid ESC chimeras were tetraploid as determined by FACS analysis (Supplementary Fig. S1). Tetraploid ESC-derived cells were chimeric in extraembryonic tissues and very small parts of fetuses at 13.5 dpc and 16.5 dpc (Supplementary Figs S2 and S3B).

### Comparison of the diploid and tetraploid ESCs proliferation and differentiation

**Figure 4:**
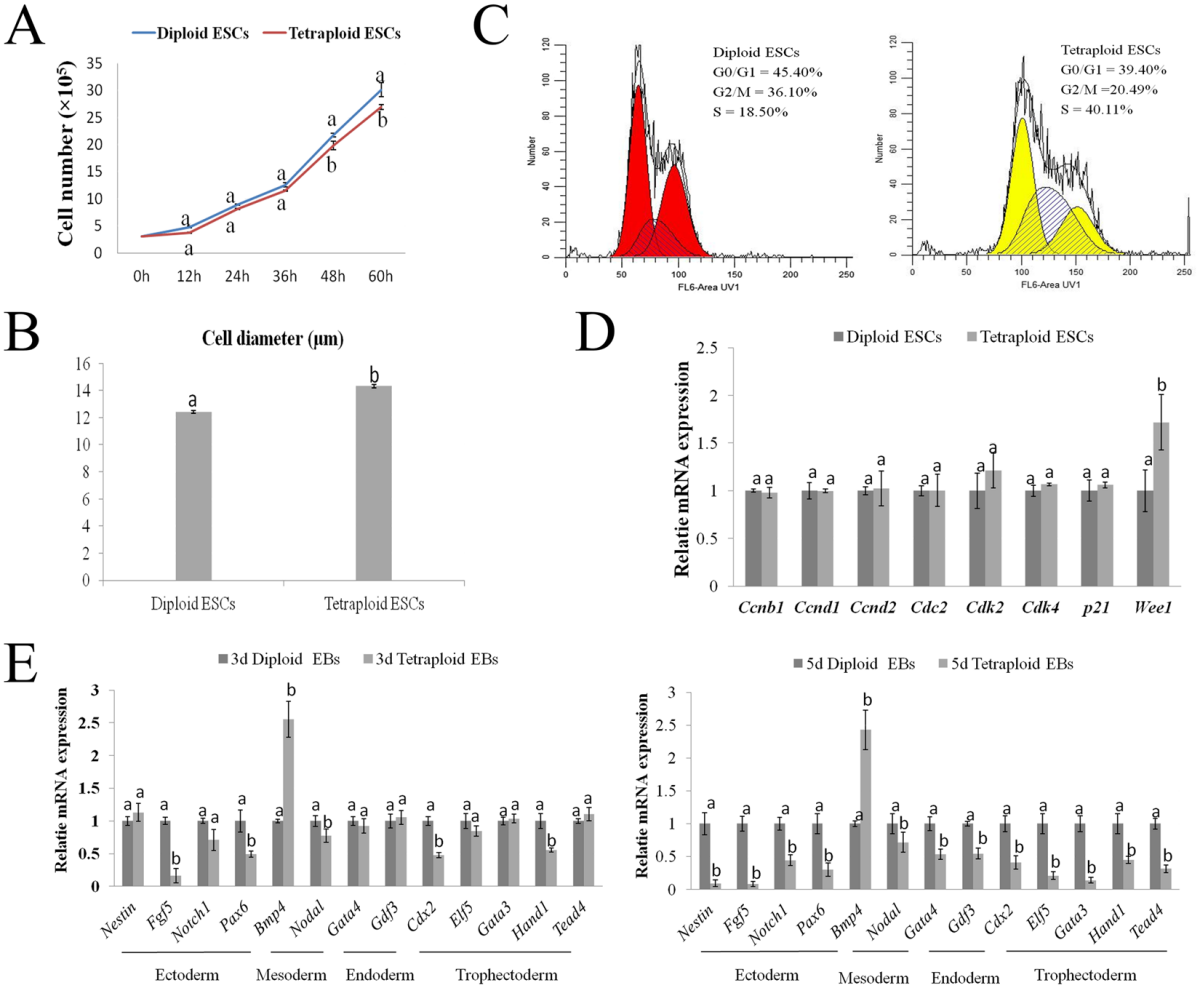
Proliferation and differentiative potential of diploid and tetraploid ESCs. (**A**) Proliferation of diploid and tetraploid ESCs. (**B**) Cell diameter of diploid and tetraploid ESCs. (**C**) FACS analysis of cell cycle differences between diploid and tetraploid ESCs. (**D**) Relative mRNA expression levels of cell division-related genes in diploid and tetraploid ESCs. (**E**) Relative mRNA expression levels of three germ layer and trophectoderm genes in 3 d and 5 d diploid and tetraploid EBs. Different superscripts represent statistically significant differences between groups (*P* < 0.05).

Approximately 3 × 10^5^ diploid or tetraploid ESCs were cultured in 6-well plates. At 12 h, 24 h and 36 h, there was no significant difference in the proliferation between diploid and tetraploid ESCs, but a difference in proliferation was observed between the two types of ESCs at 48 h and 60 h (Fig. 4A). In addition, the cell diameter of tetraploid ESCs was bigger than that of diploid ESCs by approximately 2 μm (Fig. 4B). By FACS analysis, the percentage of tetraploid ESCs in S phase was higher than in diploid ESCs, and the percentage of tetraploid ESCs in G2 phase was lower than in diploid ESCs (Fig. 4C). By quantitative real-time PCR, the relative expression of *Wee1*, which is a key gene related to the cell mitosis, was significant higher in tetraploid ESCs than that in diploid ESCs (*P* < *0*.*05*) (Fig. 4D). To further study the differentiation capacity of diploid and tetraploid ESCs, we analyzed the expression of three germ layer (i.e., ectoderm, mesoderm and endoderm)-specific genes and trophectoderm-specific genes of EBs on the third day (3 d) and the fifth day (5 d) (Fig. 4E). As shown in Fig. 4E, the relative expression of most three germ layer-specific genes and trophectoderm-specific genes of the tetraploid EBs were lower (*P* < *0*.*05*) at 3 d and 5 d, but the mesoderm-specific gene *Bmp4* was significantly higher (*P* < *0*.*05*) than that of diploid EBs at 3 d and 5 d. These indicated that the differentiation of tetraploid ESCs is defective when compared with diploid ESCs.

## Discussion

In present study, we established mouse tetraploid ESC lines from 4.5 dpc tetraploid blastocysts and obtained *in vivo* chimeras by injecting tetraploid ESCs into mouse diploid embryos. Our results showed that tetraploid ESCs maintained intrinsic pluripotency and differentiation potential during postimplantation development *in vivo*. In early mouse development before the morula stage, each blastomere within the embryo is considered to be fully totipotent. However, at the late blastocyst stage, the ICM is committed to the development of primitive endoderm, primitive ectoderm and part of the trophectoderm^25^. Tetraploid ESCs derived from the ICM of tetraploid blastocysts were injected into 4–8 cell embryos, providing an interesting tool to study mouse development; furthermore, tetraploid ESC chimeras could be used as a model to study human mosaic polyploid embryos.

Some studies have proven that mouse tetraploid embryos develop normally before implantation^8, 26–28^. The total cell number in tetraploid blastocysts was lower than that in diploid blastocysts^29^. Our results showed the NANOG was expressed in the epiblast of 4.5 dpc tetraploid embryos, which was similar to previous reports^30^, and the *Oct4* promoter of 4.5 dpc tetraploid blastocysts had low methylation levels, which was similar to diploid 4.5 dpc blastocysts. However, the epiblast cells of tetraploid blastocysts and the percentage of epiblast cells in 4.5 dpc blastocysts was significantly lower than in diploid blastocyst. The efficiency of tetraploid embryo outgrowth formation was lower than that of diploid embryo outgrowth formation. This suggested that the reduced number of epiblast cells in tetraploid embryos was one of the reasons that tetraploid blastocysts had low efficiency of outgrowth formation. In addition, the tetraploid ESCs exhibited positive AP staining, pluripotent gene expression, teratoma formation and differentiation just as the diploid ESCs.

We produced chimeras by injecting tetraploid ESCs into 4–8 cell diploid embryos. In previous studies, tetraploid ESCs contributed to the ICM in blastocysts^6^, which was consistent with our results. Tetraploid compensation studies have shown that all trophoblasts and parts of primitive endoderm derivatives cells such as the placental trophoblast and extraembryonic endoderm (including the yolk sac endoderm) were derived from tetraploid embryos, and diploid ESCs fully retained the potential of primitive ectoderm lineage to contribute to the yolk sac mesoderm, amniotic membrane, embryo allantois and umbilical cord. ESCs retained the developmental limitations of their original origin, and diploid ESCs did not contribute to the primitive endoderm and trophoblast cell lines during the chimeras’ development, therefore these ESCs did not contribute to the extraembryonic endoderm and placental trophoblast^10^. Our results indicated that tetraploid ESCs retained locational potential to remain in the correct location for development of the embryonic lineage similar to diploid ESCs; however, chimeric and survival capacities of tetraploid ESCs were poorer than diploid ESCs *in vivo* at 6.5 dpc and 8.0 dpc. At 10.5 dpc, tetraploid cells were found in the placenta, embryolemma, umbilical cord and fetuses, just as in the diploid ESC chimeras; however, the survival and chimeric capacities were poorer than in diploid ESC chimeras as observed at 6.5 dpc and 8.5 dpc. A previous study showed that tetraploid ESCs derived from somatic cell nuclear transfer did not contribute to implanted embryos^28^. In addition, the hybrid tetraploid ESCs can contribute to the embryos at 7.5 dpc, however, the chimeric parts was very poor^3^. In our study, the chimeric parts were larger at 6.5 dpc and 8.0 dpc, but the development of most chimeras were abnormal. In addition, previous results has showed that the chimeric efficiency of 2nESC chimeras which inject 2nESCs into 4–8 cells embryos was higher than that of blastocyst^31, 32^, we thought that might be led by the different injection method which we used the 4–8 cells embryos and the provious study was blastocysts. As the embryogenesis progressed, GFP cells were found in the amniotic membrane, umbilical cord and placenta at 13.5 dpc and 16.5 dpc but very less GFP cells were found in the fetus, therefore, we speculate that the tolerance of the extraembryonic tissues to the polyploid is stronger than that of the embryos. There were reports of the birth of chimeras generated by the injection of tetraploid hybrid cells into diploid blastocysts live to adulthood^16–18^, however, in our study, we detected that the chimeric parts were smaller and smaller from 13.5 dpc to 16.5 dpc. we thought that might be led by the different injection method just as the stage from 6.5 dpc to 10.5 dpc.

The proliferation of tetraploid ESCs was slower than that of diploid ESCs, which was similar to previous reports^6^. Our study further showed that the percentage of tetraploid ESCs in G2 phase was lower than that in diploid ESCs, and another study had shown that a higher percentage of cells in G2 phase was favorable for cell proliferation^33^. The percentage of tetraploid ESCs in the S phase was higher than that in diploid ESCs. It is possible that tetraploid ESCs need more time to replicate DNA to protect the normal development of cells. In addition, mammalian studies have shown that cells with a volume diameter smaller than 80% of normal cells could not undergo mitosis; one important contributor to this is Wee1 protein kinase inhibition of Cdc2 protein activity, which inhibits mitosis^34^. In our study, the relative expression of *Wee1* in tetraploid ESCs was significantly higher than in diploid ESCs, and the tetraploid ESC diameter was larger than diploid ESCs by approximately 2 μm, which indicates that tetraploid ESCs might be need more Wee1 protein to inhibit Cdc2 protein activity to promote cell volume growth; this could reduce the proliferation of tetraploid ESCs. Therefore, we speculate that the proliferation of tetraploid cells in chimeras could not keep pace with the growth of diploid cells. The tetraploid ESC chimeras could survive if the mouse has enough diploid cells to preserve the development of the chimeras. In contrast, the tetraploid ESC chimeras would not survive if the mouse has fewer diploid cells. In addition, compared with diploid ESCs, tetraploid ESCs showed that the relative mRNA expression of most triploblastic and trophoblast genes was lower and significantly lower in tetraploid EBs at 3 d and 5 d, respectively; however, the mesoderm differentiation gene *Bmp4* in tetraploid EBs was significantly higher than that in diploid EBs at 3 d and 5 d. This shows that the tetraploid and diploid ESCs can differentiate into three germ layer cells; however, its differentiation potential was not identical compared with diploid ESCs.

In the current study, chimeras produced by injecting tetraploid ESCs into diploid embryos could undergo postimplantation development. Tetraploid ESCs showed a similar distribution throughout the chimeras as diploid ESCs, but their chimeric and survival capacities were poor. Our results show that except the reason of tetraploid ESC aneuploidy chromosome, slower proliferation and disordered differentiation potential of tetraploid ESCs compared with diploid ESCs might be two of the reasons that lead to the abnormal development of tetraploid ESC chimeras.

## Materials and Methods

Unless otherwise indicated, all chemicals and media were purchased from Sigma-Aldrich. All methods were performed in accordance with the relevant guidelines and regulations. All animal handling procedures were reviewed and approved by the Animal Care and Use Criterion of China Agriculture University and were performed in accordance with the SKLAB (State Key Laboratory for Agrobiotechnology) Animal Study Proposal (SKLAB-2016-01-04).

### Mice

All mouse purchased from Vital River of China were maintained on a constant light-dark cycle (06:00 a.m.–06:00 p.m. light, 06:00 p.m.–6:00 a.m. dark). Animals were provided with commercial pelleted food.

### Embryo culture

All embryo culturing was performed in microdrops on standard bacterial petri dishes (Nunc Roskilde, Denmark) under mineral oil. M2 media (Millipore) was used for room temperature operations whereas long-term culture was conducted in bicarbonate-buffered KSOM (Millipore) in a 37 °C incubator containing 5% CO_2_.

### Production of tetraploid embryos

GFP strain 129 females (six to eight weeks old) were superovulated by injections of 5 IU of pregnant mare serum gonadotropin (Sansheng, Ningbo, China) at 5:00 p.m. followed by 5 IU of human chorionic gonadotropin (Sansheng, Ningbo, China) 48 h later, after which these females were mated with corresponding males (eight to ten weeks old). The presence of a vaginal plug the next morning was taken as evidence of mating and this was defined as 0.5 dpc of gestation. On the afternoon of the second day of gestation, the oviducts of females were flushed with M2 media to recover late two-cell embryos. The two-cell embryos were placed between two platinum electrodes 1 mm apart in a nonelectrolyte solution containing 0.3 M mannitol, 0.1 mM calcium chloride, 0.1 mM magnesium sulfate, and 0.3% BSA in the electrode chamber (Microslide450-1, BTX Inc, San Diego, CA, USA). The blastomeres were fused by two short electric pulses (100 V for 50 μsec) applied by an Electro Cell Manipulator (ECM2001, BTXInc.)^35^.

### Establishment of Diploid and tetraploid ESCs

Diploid or tetraploid blastocysts 4.5 dpc were transferred to a gelatin (Millipore)-coated cell culture dish with mitomycin-C-treated mouse embryonic fibroblast (MEF) feeder cells and cultured in stem cell medium comprising DMEM (Gibco) with 20% FBS (Gibco), 1% NEAA (Gibco), 1% GlutaMAX-L (Gibco) and 1% penicillin/streptomycin (Gibco); 0.1 mM b-mercaptoethanol; and 1,000 units/ml ESGRO leukemia inhibitory factor (LIF; Millipore). After 6–7 days, the colonies were digested with TrypLE (Invitrogen), transferred to a new gelatin-coated cell culture dish with mitomycin C-treated MEFs, and cultured in fresh medium; these cells were designated as P1. Then, the ESCs from P2 were cultured in stem cell medium comprising DMEM with 15% FBS, 1% NEAA, 1% GlutaMAX-L, 1% penicillin/streptomycin, 0.1 mM b-mercaptoethanol, 1 μM PD0325901 (Selleck), 3 μM CHIR99021 (Selleck) and 1,000 units/ml LIF.

### Alkaline Phosphatase staining and Karyotype analysis

The alkaline phosphatase (AP) activity of tetraploid ESCs was determined using an Alkaline Phosphatase Detection Kit (Millipore) according to the manufacturer’s instructions. For karyotype analysis, after centrifugation at 1500 r/min for 5 min, metaphase chromosomes were prepared by exposing cultured cells to KaryoMAX Colcemid Solution (Gibco) for 3 h followed by hypotonic treatment in 0.075 mol/L KCl for 20 min at 37 °C and fixation with cold methanol: glacial acetic acid (3:1) solution for 15 min. The samples were then dropped on cold slides, dried at room temperature, and stained with 10% Giemsa. The images were captured on a Nikon A1 microscope.

### Immunocytochemistry

For immunocytochemical analysis, embryos and ESCs were fixed with 4% paraformaldehyde (PFA) in DPBS for 20 min at room temperature. Fixed embryos and ESCs were washed three times with DPBS, incubated in 0.2% Triton X-100 buffer for 15 min, and washed three times with DPBS. After blocking in 2% BSA blocking buffer for 1 h, embryos and ESCs were incubated at 4 °C overnight in 1% BSA buffer containing primary antibodies. The following primary antibodies were used: anti-OCT4 (Santa Cruz), anti-SOX2 (Abcam), anti-SSEA1 (Cell Signaling Technology), and anti-NANOG (Cell Signaling Technology). The embryos and ESCs were washed in DPBS and incubated for 1 h with secondary antibody. For nuclear staining, the cells were incubated for 2 min with Hoechst 33342 (10 ng/ml) (Life Technologies). The images were captured using Nikon microscope.

### EBs and teratoma formation assay

The diploid and tetraploid ESCs were dissociated with TrypLE into a single cell suspension, seeded into a 6-well plate and cultured in embryoid body forming media consisting of DMEM with 10% FBS, 1% NEAA, 1% GlutaMAX-L and 1% penicillin/streptomycin. The 6-well plates were placed on a shaker (40 r/min) in a 37 °C incubator containing 5% CO_2_. For teratoma formation, tetraploid ESCs (2 × 10^7^) were subcutaneously injected into the BALB/c nude mice. Four weeks after injection, the mice were euthanized by carbon dioxide (CO_2_) inhalation, and the resulting teratomas were excised, fixed in 4% PFA, and embedded in paraffin; subsequent sections were treated with hematoxylin and eosin stain.

### Embryo injection and Embryo transfer

Fifteen diploid and tetraploid ESCs were injected into 4–8 cell stage CD-1 embryos by micromanipulation. The injected embryos were cultured in KSOM medium until 3.5 dpc, and six to eight of the injected blastocysts were transferred into each uterine horn of 2.5 dpc pseudopregnant CD-1 females. Fetuses and extraembryonic tissues were acquired from these pregnant CD-1 mouse which were euthanized by CO_2_ inhalation.

### RNA Purification and Quantitative Real-Time PCR

Total cellular RNA of either 50 EBs or 10^6^ ESCs was extracted using an RNeasy Mini Kit (QIAGEN). Reverse transcription was performed using an oligo-dT primer and M-MLV Reverse Transcriptase (Promega). Quantitative RT-PCR analyses were performed using the LightCycler 480 SYBR Green I Master Kit (Roche) and detected with LightCycler 480II (Roche). The data were analyzed using the comparative CT (2^−ΔΔCT^) method. The ΔCT was calculated using *Actin* as an internal control. All experiments were performed with more than three biological replicates. The sequences of the PCR primers are listed in Supplementary Table S5.

### Bisulfite Sequencing

Blastocysts and ESCs were directly subjected to bisulfate conversion by using the EZ DNA Methylation Direct Kit (Zymo Research). For each group, the number of blastocysts used for bisulfite conversion each time was approximately 50, and the number of ESCs was 10^6^. Nested PCR was performed using either a methylation-specific DNA polymerase (Tiangen) or HotMaster DNA polymerase (Tiangen). The sequences of the PCR primers are listed in Supplementary Table S6. To confirm the DNA methylation state, bisulfite PCR-mediated restriction mapping was performed as previously described^36^.

### Cell proliferation analysis

Diploid or tetraploid ESCs were plated at 3 × 10^5^ cells/well in 6-well plates to examine growth curves. The cells were washed with DPBS, treated with TrypLE, and counted with a LUNA™ Automated Cell Counter at the indicated times.

### FACS analysis

The diploid and tetraploid ESCs were washed in DPBS, dissociated with TrypLE into a single cell suspension, incubated for 1 h with 10 ng/ml Hoechst 333342 in a 37.0 °C incubator containing 5% CO_2_, and analyzed by FACS (Beckman Coulter). For analysis of cells from chimeric mouse fetuses and extraembryonic tissues, the tissues were washed in DPBS three times, digested with 0.25% Trypsin-EDTA and 0.05% collagen enzyme for 1 h on ice, subjected to repeated suction and blowing with a transfer pipettor, incubated for 1 h with 10 ng/ml Hoechst 33342 in a 37.0 °C incubator containing 5% CO_2_, and analyzed by FACS.

### Statistical analysis

Each experiment was repeated three times, and the results are presented as the mean ± standard error. Data were analyzed by one-way analysis of variance (ANOVA) by LSD tests and χ^2^ using SPSS software. *P* < *0*.*05* was considered to be statistically significant.

## Electronic supplementary material

Supplementary data
