# Supplementary material for: Tetraploid embryonic stem cells can contribute to the development of chimeric fetuses and chimeric extraembryonic tissues
Source: Sci Rep. 2017 Jun 8;7:3030. doi: 10.1038/s41598-017-02783-0 (PMC5465063; doi:10.1038/s41598-017-02783-0)
Supplement: Supplementary file 1 — Supplementary data [file 41598_2017_2783_MOESM1_ESM.doc]

**Tetraploid embryonic stem cells can contribute to the development of chimeric fetuses and chimeric extraembryonic tissues**

**Authors:** Bingqiang Wen^a,1^, Ruiqi Li^b,1^, Keren Cheng^c^, Enhong Li^a^, Shaopeng Zhang^a^, Jinzhu Xiang^a^ , Yanliang Wang^a^, Jianyong Han^*a^

**Author affiliations:**

^a^ State Key Laboratory for Agro biotechnology, College of Biological Sciences, China Agricultural University, Beijing 100193, People’s Republic of China

^b^ Reproductive Medicine Centre, Department of Obstetrics and Gynecology, Sun Yat-Sen Memorial Hospital, Sun Yat-Sen University, Guangzhou, China 510120, People’s Republic of China

^c^ Department of Biology, The University of Texas at San Antonio, UTSA one Circle, San Antonio, TX 78249

*Correspondence and requests for materials should be addressed to J.H. (email: [hanjy@cau.edu.cn](mailto:hanjy@cau.edu.cn))

^1^Both authors contributed equally to this work.

Supplementary Table S1-1. The chimeric capacity of diploid and tetraploid ESCs-1

at 3.5 dpc and 4.5 dpc

| Type of ESCs | No. of embryos | 3.5 dpc chimeric  Blastocysts(%) | 4.5 dpc chimeric blastocysts(%) |
| --- | --- | --- | --- |
| Diploid ESCs-1 | 58 | 58 (100%) ^a^ | 58 (100%) ^a^ |
| Tetraploid ESCs-1 | 61 | 61 (100%) ^a^ | 61 (100%) ^a^ |

Supplementary Table S1-2. The chimeric capacity of diploid and tetraploid ESCs-2

at 3.5 dpc and 4.5 dpc

| Type of ESCs | No. of embryos | 3.5 dpc chimeric  Blastocysts(%) | 4.5 dpc chimeric blastocysts(%) |
| --- | --- | --- | --- |
| Diploid ESCs-2 | 46 | 46 (100%) ^a^ | 46 (100%) ^a^ |
| Tetraploid ESCs-2 | 43 | 43 (100%) ^a^ | 43 (100%) ^a^ |

Supplementary Table S2-1. The chimeric and survival capacities of diploid and

tetraploid ESCs-1 chimeras at 6.5 dpc

| Type of ESCs | No. of transplanted chimeric embryos | No. of surviving embryos(%) | No. of chimeras (%) |
| --- | --- | --- | --- |
| Diploid ESCs-1 | 21 | 17 (80.95%) ^a^ | 17 (100%) ^a^ |
| Tetraploid ESCs-1 | 21 | 9 (42.86%) ^b^ | 8 (88.89%) ^a^ |

Supplementary Table S2-2. The chimeric and survival capacities of diploid and

tetraploid ESCs-2 chimeras at 6.5 dpc

| Type of ESCs | No. of transplanted chimeric embryos | No. of surviving embryos(%) | No. of chimeras (%) |
| --- | --- | --- | --- |
| Diploid ESCs-2 | 24 | 21 (87.50%) ^a^ | 19 (90.48%) ^a^ |
| Tetraploid ESCs-2 | 24 | 11 (45.83%) ^b^ | 7 (63.63%) ^a^ |

Supplementary Table S3-1. The chimeric and survival capacities of diploid and

tetraploid ESCs-1 chimeras at 8.0 dpc

| Type of ESCs | No. of transplanted chimeric embryos | No. of surviving embryos(%) | No. of chimeras(%) |
| --- | --- | --- | --- |
| Diploid ESCs-1 | 20 | 14 (70.00%) ^a^ | 11 (78.57%) ^a^ |
| Tetraploid ESCs-1 | 20 | 9 (45.00%) ^a^ | 4 (44.44%) ^a^ |

Supplementary Table S3-2. The chimeric and survival capacities of diploid and

tetraploid ESCs-2 chimeras at 8.0 dpc

| Type of ESCs | No. of transplanted chimeric embryos | No. of surviving embryos(%) | No. of chimeras(%) |
| --- | --- | --- | --- |
| Diploid ESCs-2 | 19 | 18 (94.74%) ^a^ | 15 (83.33%) ^a^ |
| Tetraploid ESCs-2 | 20 | 12 (60.00%) ^b^ | 5 (41.67%) ^b^ |

Supplementary Table S4-1. The chimeric and survival capacities of diploid and

tetraploid ESCs-1 chimeras at 10.5 dpc

| Type of ESCs | No. of transplanted chimeric embryos | No. of surviving embryos(%) | No. of chimeras(%) |
| --- | --- | --- | --- |
| Diploid ESCs-1 | 27 | 19 (70.37%) ^a^ | 17 (89.47%) ^a^ |
| Tetraploid ESCs-1 | 28 | 10 (35.71%) ^b^ | 3 (30.00%) ^b^ |

Supplementary Table S4-2. The chimeric and survival capacities of diploid and

tetraploid ESCs-2 chimeras at 10.5 dpc

| Type of ESCs | No. of transplanted chimeric embryos | No. of surviving embryos(%) | No. of chimeras(%) |
| --- | --- | --- | --- |
| Diploid ESCs-2 | 24 | 19 (79.17%) ^a^ | 14 (73.69%) ^a^ |
| Tetraploid ESCs-2 | 24 | 11 (45.83%) ^b^ | 4 (36.36%) ^b^ |

Supplementary Table S5. Primers for qRT-PCR

| Genes | Forward primer sequence (5' to 3') | Reverse primer sequence (5' to 3') |
| --- | --- | --- |
| *Actb* | GATCTGGCACCACACCTTCT | GGGGTGTTGAAGGTCTCAAA |
| *Essrb* | TTTCTGGAACCCATGGAGAG | AGCCAGCACCTCCTTCTACA |
| *Fn1* | GAAGTCGCAAGGAAACAAGC | GTTGTAGGTGAACGGGAGGA |
| *Klf2* | ACCAAGAGCTCGCACCTAAA | GTGGCACTGAAAGGGTCTGT |
| *Nanog* | TTCTTGCTTACAAGGGTCTGC | AGAGGAAGGGCGAGGAGA |
| *Nr5a2* | TGCTGAGCCCTGAAGCTATT | AGGGTTACTGCCCGTTTTCT |
| *Pou5f1* | GTTGGAGAAGGTGGAACCAA | CTCCTTCTGCAGGGCTTTC |
| *Sox2* | AAGGGTTCTTGCTGGGTTTT | AGACCACGAAAACGGTCTTG |
| *Utf1* | CTACGAGGTTCCTTCGACCA | GACTGGGAGTCGTTTCTGGA |
| *Stella* | GAGAAGACTTGTTCGGATTGAGC | CATCGTCGACAGCCAGGG |
| *Ccnb1* | TGCATTCTCTCAGTGCCCTCCACA | AGACAGGAGTGGCGCCTTGGT |
| *Ccnd1* | CTGACACCAATCTCCTCAACG | TCCTCACAGACCTCCAGCAT |
| *Ccnd2* | CCTTTGACGCAGGCTCCCTTCT | ACCCTGGTGCACGCATGCAAA |
| *Cdc2* | AGAAGGTACTTACGGTGTGGT | GAGAGATTTCCCGAATTGCAGT |
| *Cdk2* | TCCCTGTCCGAACTTACACTC | GGGTCACCATTTCAGCAAA |
| *Cdk4* | AGAAACCCTCGCTGAAGCGGCA | TGGGGGTGAACCTCGTAAGGAGA |
| *P21* | CCTGGTGATGTCCGACCTG | CCATGAGCGCATCGCAATC |
| *Wee1* | ACAGTCGTATGTGCTGCTGG | CTTCTCTCTGGGTCAGGATGA |
| *Nestin* | GATCGCTCAGATCCTGGAAG | AGGTGTCTGCAAGCGAGAGT |
| *Fgf5* | ATCTACCCGGATGGCAAAGT | TCTCGGCCTGTCTTTTCAGT |
| *Notch1* | GGCACAACTCCACTGATCCT | CAGCATCCACATTGTTCACC |
| *Pax6* | AACAACCTGCCTATGCAACC | ACTTGGACGGGAACTGACAC |
| *Bmp4* | AGGAGGAGGAGGAAGAGCAG | CCTGGGATGTTCTCCAGATG |
| *Nodal* | ACGTTCACCGTCATTCCTTC | GTAGGGCTGATGCCAACACT |
| *Gata4* | TCTCACTATGGGCACAGCAG | GCGATGTCTGAGTGACAGGA |
| *Gdf3* | ACCTTTCCAAGATGGCTCCT | ACAGAGCAACCACCAGCTCT |
| *Cdx2* | AAGACAAATACCGGGTGGTG | CCAGCTCACTTTTCCTCCTG |
| *Elf5* | CCCTCCTCCTCTTCAAAACC | AAGTTGCCACAAGACCATCC |
| *Gata3* | CTTATCAAGCCCAAGCGAAG | CATTAGCGTTCCTCCTCCAG |
| *Hand1* | TCAAAAAGACGGATGGTGGT | GCGCCCTTTAATCCTCTTCT |
| *Tead4* | AAACTGTGGACATCCGCCAAATC | ATGTTCTCCGGGCTCTCGTACTG |

Supplementary Table S6. Primers for bisulfite PCR

| Gene | Primer type | Primer sequence (5’) |
| --- | --- | --- |
| *Oct4* | Out forward | GAGTATTTAGGAGGTATAAGAATT |
|  | Out reverse | ATCAAAAACTAACATAAACCCCT |
|  | In forward | GTAAGGAGATTATGTTTATTTTTGG |
|  | In reverse | CTAACCTCATAAAACCCATAACTAT |


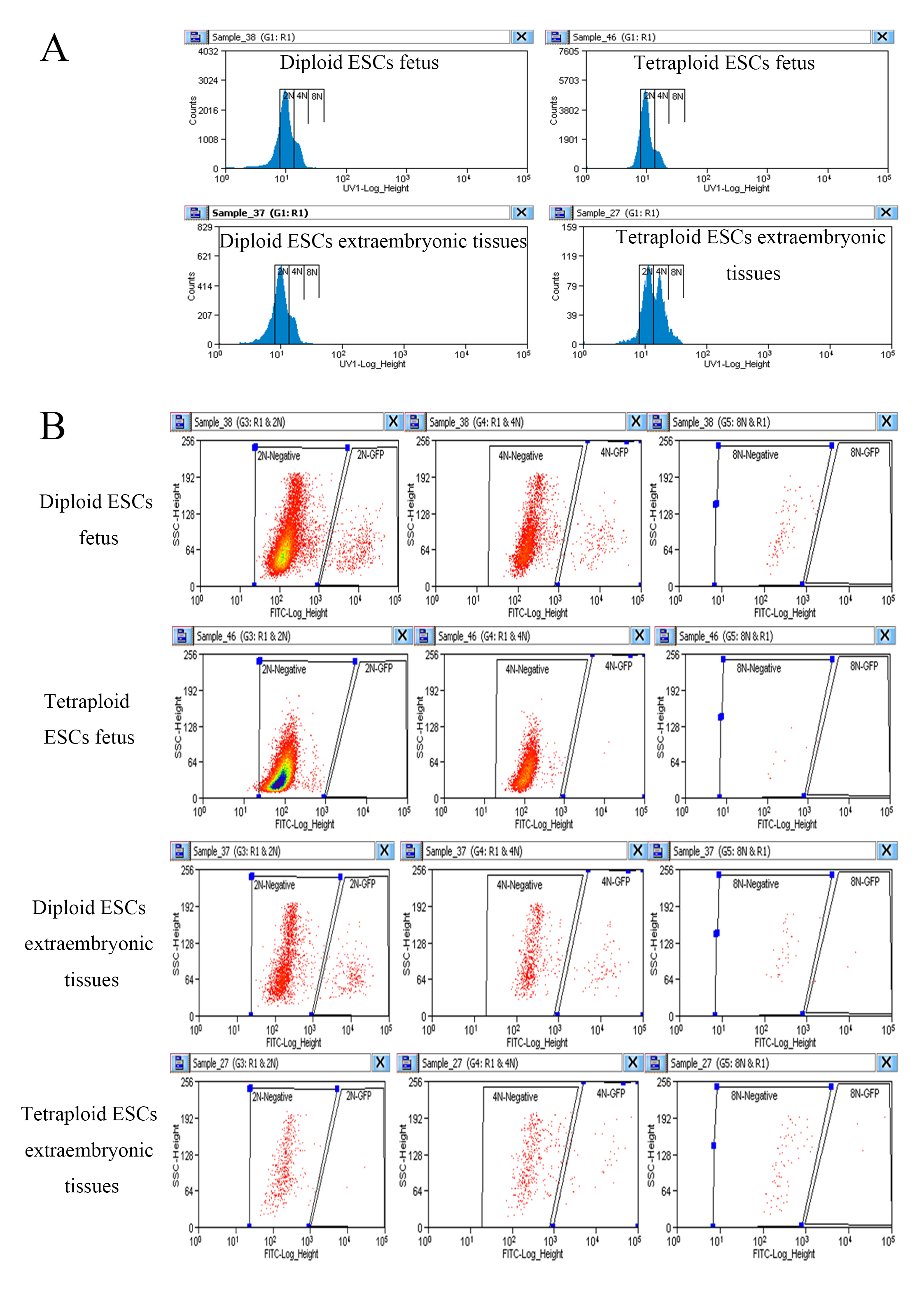


**Supplementary Figure S1. FACS analysis of cell ploidy and cell numbers in chimeras**

(A) FACS analysis of cell ploidy in chimeric fetuses and the extraembryonic tissues of diploid and

tetraploid ESCs.

(B) FACS analysis of cell numbers in chimeric fetuses and extraembryonic tissues of diploid and tetraploid ESC chimeras.

2N: Two sets of chromosomes, 4N: Four sets of chromosomes, 8N: Eight sets of chromosomes,

Negative: Cells not expressing GFP, GFP: cells expressing GFP.


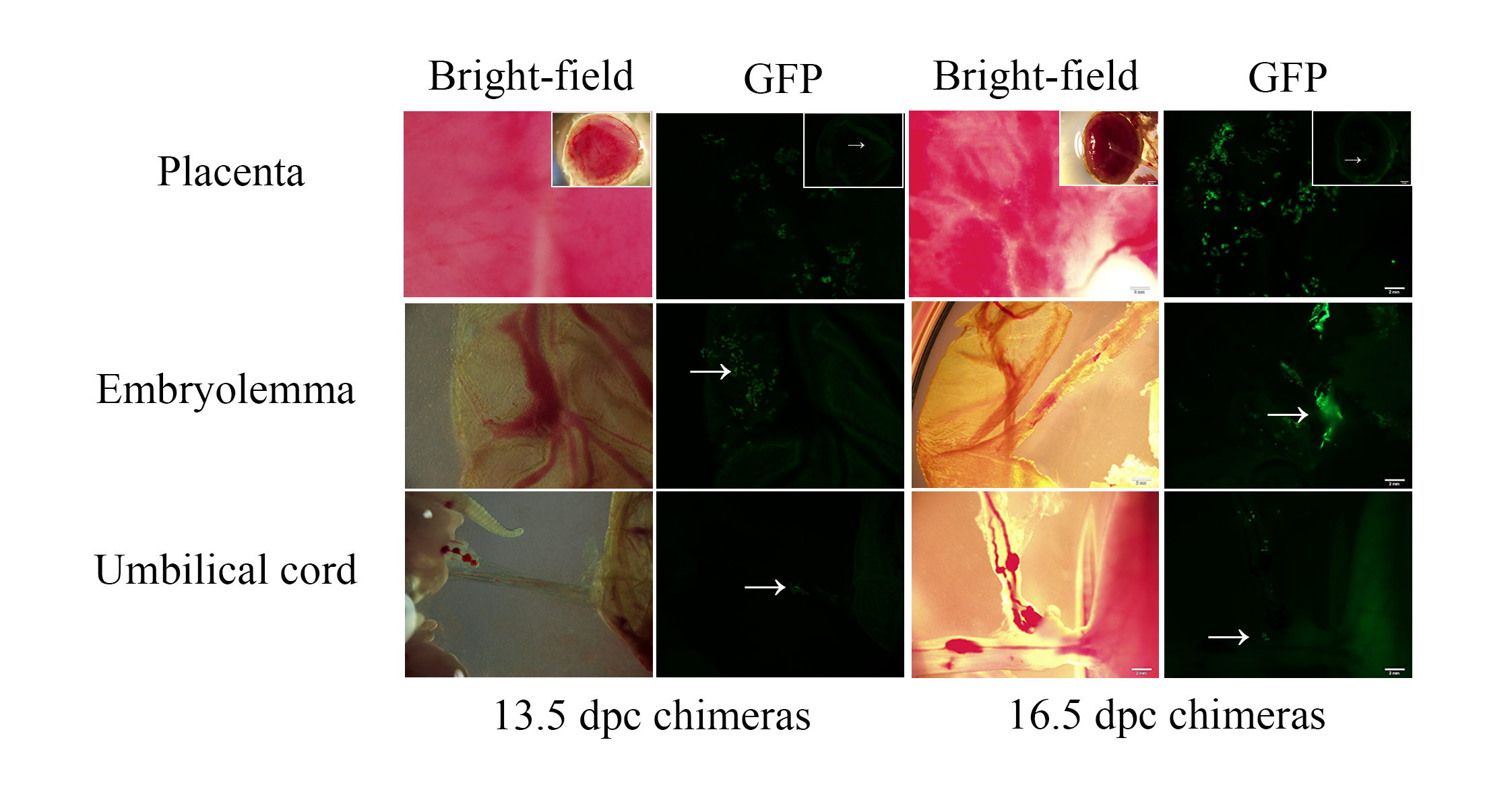


**Supplementary Figure S2. Chimeric extraembryonic tissues of tetraploid ESCs-1.**

Chimeric extraembryonic tissues of tetraploid ESCs-1 at 13.5 dpc and 16.5 dpc. The arrow indicates the parts comprising chimeric cells.

**Supplementary Figure S3. Chimeras of diploid ESCs-2 and tetraploid ESCs-2.**

(A) Diploid ESCs-2 and tetraploid ESCs-2 chimeras at 6.5 dpc (Bar scale = 200 μm), 8.0 dpc (Bar

scale = 200 μm) and 10.5 dpc.

(B) Diploid ESCs-2 and tetraploid ESCs-2 chimeras at 13.5 dpc and 16.5 dpc.
